# Respiratory Failure Associated With Mutations in the RYR1 Gene: A Case Report

**DOI:** 10.1002/ccr3.70971

**Published:** 2025-10-17

**Authors:** Chenliang Zhao, Yongxiang Li, Jinhui Li, Jianrong Xiong

**Affiliations:** ^1^ Department of Critical Care Medicine Heyou Hospital Foshan City Guangdong P.R. China; ^2^ Department of Rehabilitation Medicine The Fourth Affiliated Hospital of School of Medicine, and Internal School of Medicine Yiwu Zhejiang P.R. China; ^3^ Department of Chinese Medicine & Rehabilitation The Second Affiliated Hospital of Zhejiang University School of Medicine Zhejiang Hangzhou China; ^4^ Department of Rehabilitation, Xinhua Hospital, School of Medicine Shanghai Jiao Tong University Shanghai China; ^5^ Department of Rehabilitation Medicine The First Affiliated Hospital of Zhejiang Chinese Medical University (Zhejiang Provincial Hospital of Chinese Medicine) Zhejiang Hangzhou China

**Keywords:** congenital myopathy, respiratory failure, RYR1, treatment

## Abstract

A novel *RYR1* mutation (c.C5701T:p.Q1901X) was identified in a 51‐year‐old female presenting with acute respiratory failure as the primary manifestation of congenital myopathy. This case expands the genotype–phenotype spectrum of *RYR1*‐related myopathies and demonstrates that multidisciplinary intervention—including ventilator support, tracheostomy, and targeted rehabilitation—can significantly improve functional outcomes in late‐onset cases.

## 1 Introduction

Congenital myopathies (CM) are a group of early‐onset, non‐dystrophic, genetically heterogeneous disorders characterized by distinct muscle pathology. Estimates suggest that the prevalence of these disorders is approximately 1 in 26,000 [1]. The primary clinical manifestations of congenital myopathies are muscle weakness and hypotonia, with significant weakness commonly observed in the axial and proximal muscle groups [2]. Involvement of extraocular muscles, as well as cardiac and respiratory muscles, and/or distal muscles may be linked to specific genetic defects. Moreover, patients may present with spinal deformities, malignant hyperthermia, and cardiac involvement [3].

Previous studies have identified the genetic factors associated with various forms of congenital myopathies, including DNM2, NM, MTM1, MYH7, TTN, ACTA1, NEB, and SEPN1, among others [3]. Mutations in the DNM2 gene primarily present with ocular involvement, foot drop, pes cavus, facial weakness, and facial deformities. In contrast, mutations in the NM gene predominantly manifest as scoliosis, severe facial weakness, facial deformities, and pronounced congenital hypotonia [3].

Currently, research on gene therapy is highly active, encompassing numerous genetic‐related diseases. Clinical trials of Rycal S48168 (also known as ARM210) have shown promising progress. Recent research findings indicate that Rycal S48168 has potential in treating myopathies associated with Ryanodine Receptor 1 (RYR1) [4]. Researchers have confirmed the safety and tolerability of Rycal S48168 at doses of 120 and 200 mg [4]. Notably, in the 200 mg dose group, patients experienced significant relief from fatigue symptoms and improvements in proximal muscle strength [4]. Gene therapy holds considerable promise for benefiting patients with congenital myopathies [5].

In this study, we report a case of a patient presenting with respiratory failure as the primary symptom, in whom a mutation in the RYR1 gene was identified through genetic testing. Following clinical intervention, the patient was successfully transitioned from the Respiratory Intensive Care Unit to the rehabilitation ward. This case serves as a valuable reference for our clinical diagnosis and treatment practices, thereby enriching the genotype–phenotype spectrum of congenital diseases.

## 2 Case History/Examination

This study received ethical approval from the Ethics Committee (acceptance number: 2024 Section 1017‐2).

Clinical evolution: A 51‐year‐old Chinese female patient was admitted to the respiratory department with a chief complaint of cough accompanied by sputum and chest tightness, persisting for 5 days and worsening over the last day. On the second day of hospitalization, the patient suffered a sudden cardiac and respiratory arrest. Immediate cardiopulmonary resuscitation (CPR) was initiated, and endotracheal intubation was performed. Following the restoration of spontaneous circulation, the patient was transferred to the respiratory intensive care unit (RICU) for close monitoring and treatment. The patient's condition stabilized, and we attempted extubation twice; however, both attempts were unsuccessful due to the patient's diminished ability to cough and expectorate independently. After discussions with the patient's family, a tracheostomy was performed, and a size 7.5 plastic cannula was inserted, followed by the initiation of mechanical ventilation therapy. Five days later, we attempted to wean the patient off the ventilator and commenced high‐flow oxygen therapy through the tracheostomy cannula. Once the patient's condition stabilized, she was transferred to the rehabilitation ward for further treatment.

Physical examination reveals that the patient is conscious but exhibits a weak mental state and bilateral hearing impairment. The pupils are unequal in size, with the right pupil measuring approximately 3.5 cm in diameter and the left pupil approximately 3 cm in diameter. Pupillary light reflexes are brisk, and the palpebral fissures are narrowed bilaterally. The chest appears symmetrical, with coarse breath sounds auscultated bilaterally; however, no significant chest rise and fall is observed. Proximal muscle strength in the limbs is graded as 4, while distal muscle strength is graded as 3. Grip strength in both hands is diminished, tendon reflexes in the limbs are not elicited, and the Babinski sign is negative bilaterally.

## 3 Methods (Differential Diagnosis, Investigations, and Treatment)

### 3.1 Differential Diagnosis

The patient's presentation (respiratory failure, proximal muscle weakness, and myopathic EMG findings) warranted differentiation among genetic myopathies and acquired neuromuscular disorders. Key considerations included:

1. RYR1‐related myopathy (RYR1‐RM): Supporting evidence: Clinical: Hypotonia, proximal > distal weakness, respiratory insufficiency, and absent tendon reflexes align with RYR1‐RM phenotypes, particularly the late‐onset axial‐predominant subtype [6]. Genetic: Heterozygous *RYR1* mutations (c.C5701T:p.Q1901X and c.C3043T:p.R1015C) were identified. The novel truncating variant (Q1901X) likely causes loss‐of‐function, consistent with recessive RYR1‐RM [7]. Histopathology: Muscle biopsy showed myopathic changes without dystrophic features, typical of RYR1‐RM [3]. Against: Lack of malignant hyperthermia susceptibility or ophthalmoplegia, which occurs in 50% of RYR1‐RM cases [8].
2. Mitochondrial myopathy: Supporting evidence: Clinical: Respiratory failure, exercise intolerance, and hearing impairment (present in this case) are common in mitochondrial disorders (e.g., MELAS, MERRF) [9]. Laboratory: Elevated lactate (not measured here) and ragged‐red fibers on biopsy could support this diagnosis. Against: Genetic: No mtDNA mutations or deletions were detected via exome sequencing. Histopathology: Absence of COX‐negative fibers or mitochondrial proliferation on biopsy.
3. Muscular dystrophy (e.g., Limb‐girdle muscular dystrophy [LGMD]): Supporting evidence: Clinical: Progressive limb weakness and respiratory involvement occur in LGMD subtypes (e.g., LGMD2B/DYSF‐related). Against: Histopathology: No dystrophic features (e.g., fibrosis, fat infiltration) on biopsy. Genetic: No pathogenic variants in dystrophy‐associated genes (e.g., DYSF, CAPN3).
4. Other considerations: Myasthenia gravis: Rapidly progressive weakness and respiratory failure may mimic myasthenic crises. However, negative acetylcholine receptor antibodies and lack of response to pyridostigmine exclude this. Critical Illness Myopathy: History of prolonged ICU stay could contribute, but pre‐admission symptoms and genetic findings favor a primary myopathy.

### 3.2 Final Diagnosis

RYR1‐RM was confirmed based on genetic, histopathological, and clinical correlation, excluding other mimics.

Biochemical tests: (April 21, 2024) Blood gas analysis: Partial pressure of carbon dioxide (PaCO_₂_) was recorded at 69.5 mmHg (↑), while the partial pressure of oxygen (PaO_₂_) was 78.4 mmHg, resulting in an alveolar‐arterial oxygen gradient of 91 mmHg (↑). Complete blood count (CBC) and C‐reactive Protein (CRP) analysis revealed a white blood cell count (WBC) of 15.3 × 10^9^/L (↑), a neutrophil percentage of 88.7% (↑), and a C‐reactive protein (CRP) level of 121.8 mg/L (↑). (June 24, 2024) Arterial blood gas analysis indicated a partial pressure of carbon dioxide (PaCO_₂_) of 51.5 mmHg (↑) and a partial pressure of oxygen (PaO_₂_) of 79.4 mmHg, with an alveolar‐arterial oxygen gradient (A‐a gradient) of 74 (↑). The complete blood count and C‐reactive protein (CRP) analysis showed a white blood cell count (WBC) of 6.6 × 10^9^/L (↑), a neutrophil percentage of 72.2% (↑), and a C‐reactive protein (CRP) level of 41.7 mg/L (↑).

**FIGURE 1:**
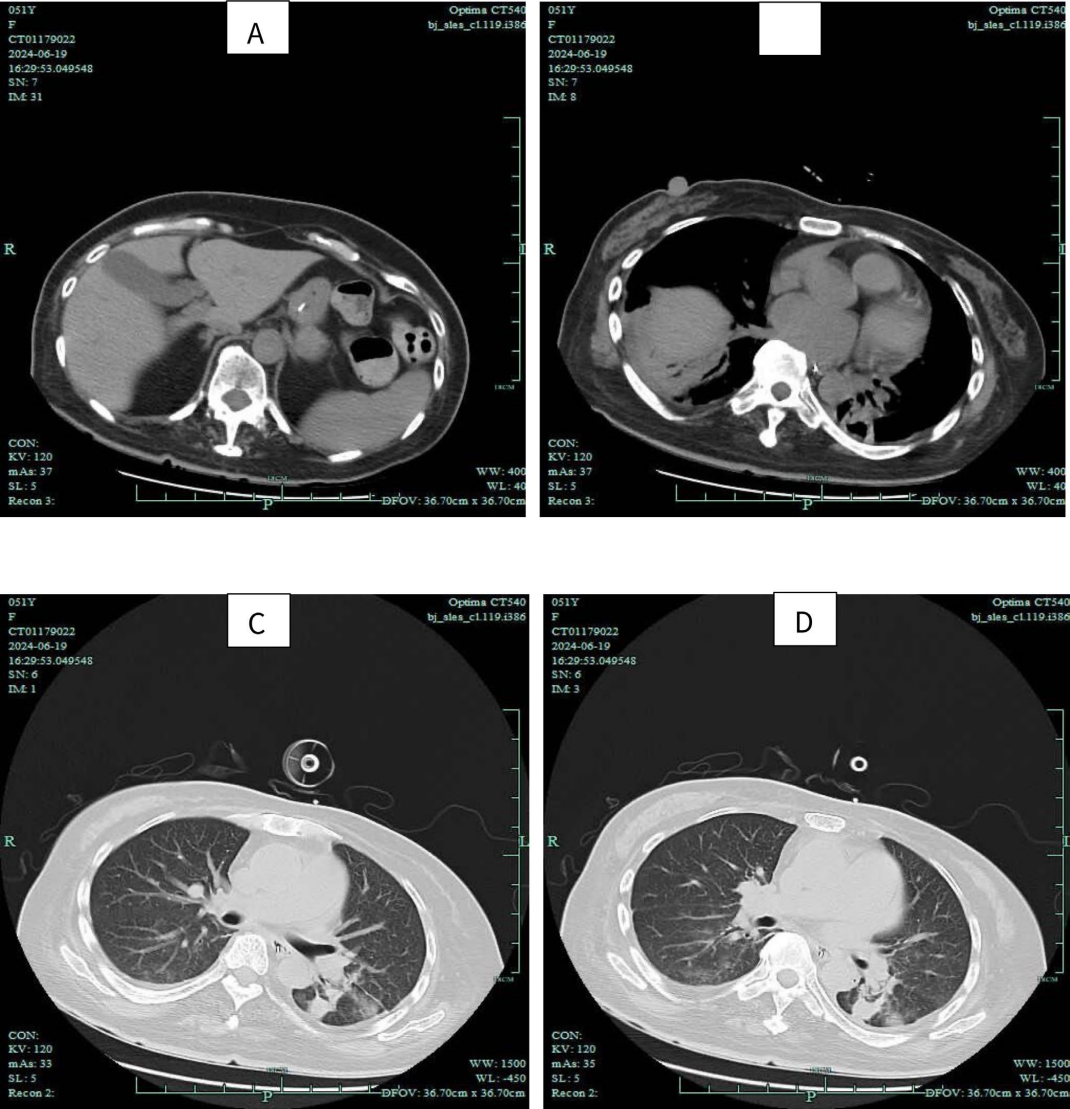
Results of noncontrast chest CT scan.

**FIGURE 2:**
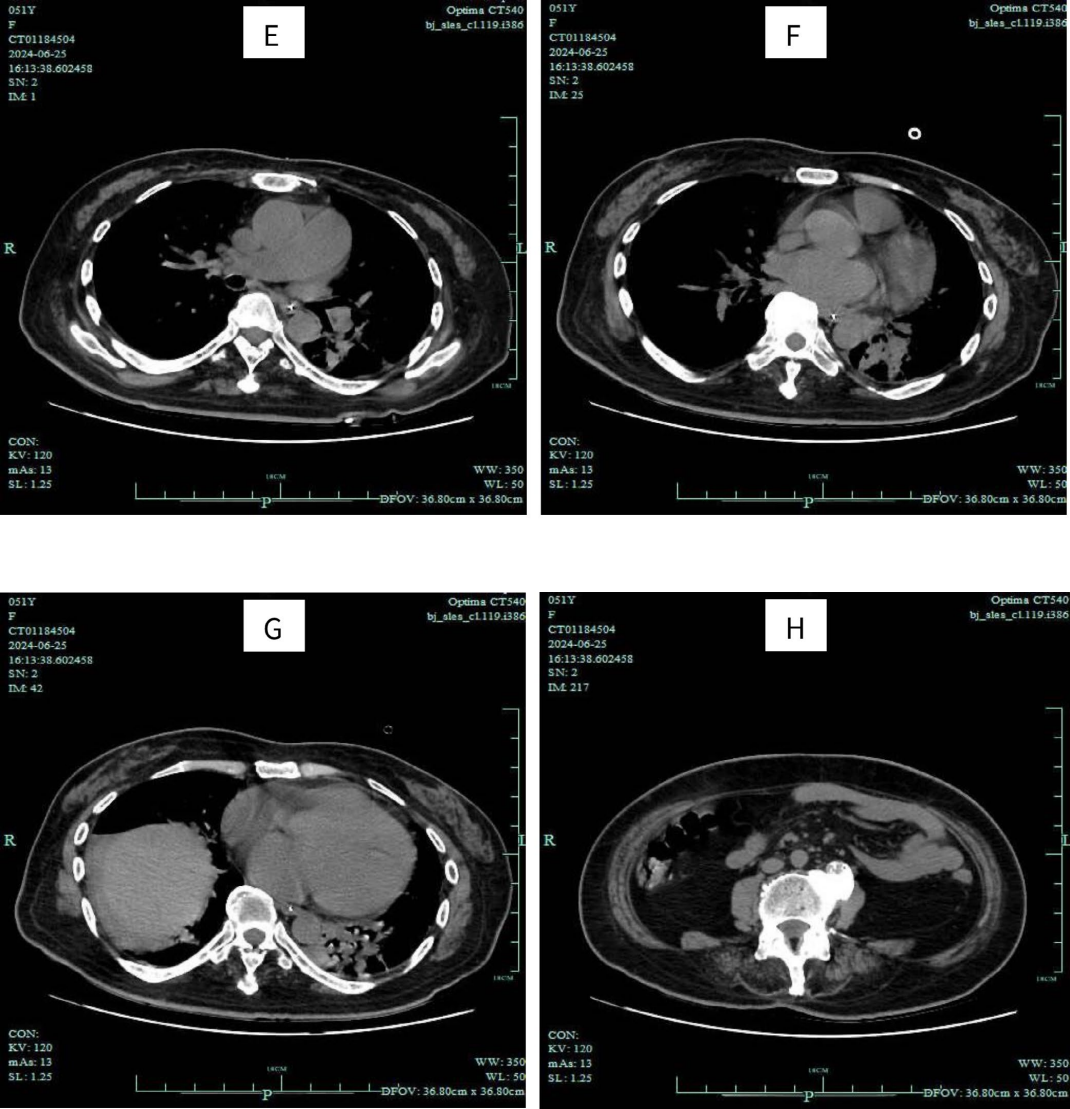
Results of noncontrast CT scan of the whole abdomen.

Auxiliary examination: Chest CT scan revealed inflammation in both lungs and atrophy of the chest wall muscles (Figure 1). Electromyography (EMG) indicated myogenic damage in the examined muscles of both the upper and lower limbs. Full Abdominal CT showed atrophy of the abdominal wall muscles (Figure 2).

Muscle biopsy demonstrated that the morphology of the skeletal muscle tissue suggests a myopathic pattern of damage.

Exome sequencing identified genetic mutations in the patient's RYR1 (c.C5701T:p.Q1901X, c.C3043T:p.R1015C) and SCN5A (c.G4282T:p.A1428S) genes through whole exome sequencing.

Respiratory medicine: The patient was treated with empirical anti‐infective therapy, consisting of Levofloxacin 0.5 g administered once daily, in conjunction with an intravenous infusion of Piperacillin‐Tazobactam 4.5 g every 8 h. Symptomatic treatment included the use of antitussive and expectorant medications. In the RICU, the patient initially underwent orotracheal intubation with ventilator support, accompanied by anti‐infective treatment, enhanced airway management, and prone ventilation therapy. Despite multiple attempts to wean the patient off the ventilator after stabilization, all efforts were unsuccessful. Following the family's consent, a tracheostomy was performed on May 24th. Subsequently, the patient received respiratory support via the tracheostomy tube, transitioning to high‐flow oxygen therapy once the condition stabilized. The patient also participated in breathing exercises and physical activity. After achieving stabilization, the patient was transferred to the rehabilitation ward.

Department of rehabilitation medicine: We continued to administer high‐flow oxygen therapy via the tracheostomy tube, enhanced oral care, and performed regular turning and back patting for the patient, while paying close attention to their positioning management. The treatment regimen included the administration of ambroxol to facilitate expectoration, nebulization with acetylcysteine and salbutamol, and the provision of nutritional support as symptomatic treatment. Rehabilitation training primarily encompassed vibration expectoration, respiratory training, motorized standing bed exercises, and physical training as integral components of comprehensive rehabilitation therapy. We initiated the process by elevating the patient's bed head, followed by bedside standing training, and subsequently, we gradually increased the patient's out‐of‐bed time.

## 4 Conclusion and Results (Outcome and Follow‐Up)

After months of treatment, the patient showed marked improvement. She regained independent mobility while receiving high‐flow oxygen therapy via tracheostomy and could communicate effectively through writing. Muscle strength improved, with proximal limbs at grade 4 and distal at grade 3. The patient was transferred to the rehabilitation ward for continued respiratory training and physical therapy. While she remained dependent on supplemental oxygen, her condition stabilized sufficiently for discharge with ongoing outpatient rehabilitation.

## 5 Discussion

Congenital myopathies are a group of rare genetic muscle disorders that typically manifest with hypotonia, muscle weakness, and skeletal deformities, either at birth or in early infancy [3, 9]. While these disorders most commonly present symptoms at birth, late‐onset forms of congenital myopathies (CM) also exist [1]. With advancements in genetic testing technologies, research has identified over 20 genes associated with congenital myopathies to date. The proteins encoded by these genes primarily participate in mechanisms such as skeletal muscle Ca^2+^ homeostasis, excitation‐contraction coupling, and the assembly and interaction of thin and thick filaments [2].

The regulation of intracellular calcium (Ca^2+^) is essential across all cell types. The ryanodine receptor (RyR), a Ca^2+^ release channel situated on the sarcoplasmic/endoplasmic reticulum (SR/ER), facilitates the release of Ca^2+^ from intracellular stores, thereby activating critical functions such as muscle contraction and neurotransmitter release. Dysfunctional RyR‐mediated Ca^2+^ handling has been linked to the pathogenesis of both inherited and non‐inherited conditions, including heart failure, cardiac arrhythmias, skeletal myopathies, diabetes, and neurodegenerative diseases [10, 11]. Furthermore, mutations in the RYR1 gene have emerged as a significant cause of non‐dystrophic myopathies, which encompass a spectrum of disease phenotypes collectively known as RYR1‐related myopathies (RYR‐RM) [6, 8].

Despite ongoing research into congenital myopathies and the continuous identification of associated genes, there remains a scarcity of clinical studies focused on the treatment of these conditions. We report a case of congenital myopathy linked to a mutation in the RYR1 gene. The patient exhibited distinctive facial features, bilateral hearing impairment, and weakness of the respiratory muscles and diaphragm, alongside generalized limb muscle weakness, which was more pronounced in the distal muscles compared to the proximal muscles. The patient underwent a comprehensive treatment regimen that included pharmacotherapy (N‐acetylcysteine, salbutamol, ambroxol injection), nursing care (regular repositioning and sputum suction), and pulmonary rehabilitation (diaphragm pacing therapy, aerobic exercise, and cough training). As a result, the patient's condition improved significantly. Research indicates that N‐acetylcysteine and salbutamol are potential therapeutic agents for RYR1‐RM and are currently in the clinical trial phase [8].

This study enhances the understanding of genotype–phenotype correlations in congenital myopathies (CM) and provides valuable insights for the clinical diagnosis and treatment of CM. However, several limitations were identified in our study. First, comprehensive genetic testing should have been performed on the patient's parents and offspring. Second, further evaluations of the patient's respiratory muscles and diaphragm should have been conducted. Third, multiple‐site muscle biopsies would have been ideal. Unfortunately, due to the patient's familial circumstances and financial constraints, we were unable to conduct a comprehensive evaluation.

## 6 Conclusions

This case report describes a congenital myopathy primarily manifesting as respiratory failure due to an RYR1 gene mutation. The patient showed significant clinical improvement following comprehensive treatment, highlighting the importance of early diagnosis and multidisciplinary management in RYR1‐related myopathies.

## Author Contributions

**Jianrong Xiong:** conceptualization, data curation, investigation, methodology, writing – original draft, writing – review and editing. **Yongxiang Li:** investigation, methodology. **Jinhui Li:** methodology, supervision. **Chenliang Zhao:** investigation, methodology, writing – review and editing.

## Ethics Statement

All procedures performed in studies involving human participants were in accordance with the ethical standards of the institutional and/or national research committee and ethical standards.

## Consent

Written informed consent was obtained from the patient for the publication of this case report and any accompanying images, in accordance with the journal's patient consent policy.

## Conflicts of Interest

The authors declare no conflicts of interest.

## Data Availability

The datasets used and/or analyzed during the present study are available from the corresponding author upon reasonable request.
